# Design of a Test for Detecting the Presence of Impulsive Noise

**DOI:** 10.3390/s20247135

**Published:** 2020-12-12

**Authors:** Hyungkook Oh, Dongho Seo, Haewoon Nam

**Affiliations:** The Division of Electrical Engineering, Hanyang University, Ansan 15588, Korea; ohhk@hanyang.ac.kr (H.O.); johnseo@hanyang.ac.kr (D.S.)

**Keywords:** impulsive noise, vehicular network, classification, Kolmogorov–Smirnov test

## Abstract

This paper proposes a new test method of detecting the presence of impulsive noise based on a complementary cumulative density function (CCDF). Impulsive noise severely degrades performance of communication systems and the conventional Kolmogorov–Smirnov (K–S) test may not perform well, because the test does not consider the characteristics of impulsive noise. In order to detect the presence of impulsive noise reliably, the CCDF of measurement samples is analyzed and compared with the CCDF of additive white Gaussian noise to find the difference between those CCDFs. Due to the nature of heavy-tails in impulsive noise, only the maximum difference may not be sufficient for the accurate detection of impulsive noise. Therefore, the proposed method applies the test hypothesis using the weighted sum of all the differences between those CCDFs. Simulation results justify that the proposed test is more robust and provides lower miss detection probability than the K–S test in the presence of impulsive noise.

## 1. Introduction

Most existing digital communication systems are designed to perform optimally in an additive white Gaussian noise (AWGN) environment, but there are some environments where impulsive noise is dominant [1,2]. For example, the performance of the vehicular network in the presence of a cellular network is limited by impulsive noise [3,4]. Since the performance of conventional communication systems is severely degraded in such an environment, detecting the presence of impulsive noise is an important task in most communication systems [5]. Upon the detection of impulsive noise, a receiver can execute a special filter, such as a non-linearity pre-processor [6,7] which is designed based on the characteristics of impulsive noise, to circumvent the impulsiveness of the noise and further achieve the optimal performance in an impulsive noise environment [8,9].

Various studies [10,11,12,13,14,15,16,17] that considered impulsive noise, including power line communications [10] and environments such as a underwater environment [11] in their conclusion. A noise detection method was researched [12,13,14,15,16,17] in an environment with impulsive noise. A previous study [12] reported the actual disturbances encountered by devices operating in one of the bands of interest during normal use. Time-domain measurement procedures are proposed to measure radiated transient interference, which is a source of distortion that offers performance degradation of wireless digital communication systems [13,14,15]. Because transient electromagnetic interference has an enormous impact on the performance of mobile communications on railways, the analysis tool of electromagnetic interference is presented based on a computation of minima so that the power ratio between transmitted signal and interference is smaller than a certain threshold [16,17], but it is difficult to adapt this scheme to a practical system since the threshold is numerically calculated.

Kolmogorov–Smirnov (K–S) test is a well-known normality test, where it quantifies the distance between the empirical cumulative distribution function (CDF) of observed samples and the reference distribution [18,19]. The K–S test is a non-parametric method to measure the goodness of a fit and the K–S detector does not require prior knowledge of the signal. To determine the noise environment and whether it is an AWGN or not, it may be suitable to employ the K–S test, but unfortunately, this test does not offer satisfactory classification about the performance when impulsive noise is present because the test does not consider the characteristics of impulsive noise. In addition, the K–S test is used for various purposes in the literature as well. In a previous study [20], a sequential K–S test was used to achieve fast spectrum sensing to identify the unused spectrum to give to an unlicensed user. The test is also used in modulation classification performed at a receiver based on received samples before demodulation [18]. Ref. [21] proposes a signal to noise ratio (SNR) estimator based on the K–S test and binary search scheme that reduces a large number of additions. Furthermore, the K–S test is used in a multiple-input multiple-output (MIMO) system for blind identification of spatial multiplexing and Alamouti space–time block code based on the correlation property of adjacent samples in [19]. In a previous study [22], the performance of normality tests for non-normal data with the various tests are compared. Their simulation results indicate that the K–S test is the most powerful test when the sample size is large (>100). However, the simulation results do not prove that the performance of K–S test is optimized for the classification of impulsive noise. Because even though the probability density of impulsive noise has heavy-tails characteristics, the K–S test utilizes only the maximum difference, not the heavy-tails characteristics of impulsive noise. If a test cannot classify the noise model correctly, the communication strategy may be incorrect; this will cause a severe degradation in communication performance.

Motivated by the aforementioned issues, this paper proposes a method to detect the presence of impulsive noise. From the received signal, the empirical complementary cumulative distribution function (CCDF) of the amplitude is computed, which explicitly shows the characteristics of impulsive noise. For comparisons, the CCDF of the amplitude under AWGN is computed in advance as a reference in advance. In order to detect the presence of impulsive noise reliably, the proposed method compares the empirical CCDF of measurement samples and the reference CCDF of AWGN to find the difference between those CCDFs. Due to the nature of heavy-tails in impulsive noise, only the maximum difference may not be sufficient for the accurate detection of impulsive noise. Thus, the proposed method applies the test hypothesis using the weighted sum of all the differences between those CCDFs. Middleton class A noise and symmetric α stable (SαS) noise models are considered for the models of impulsive noise. Simulation results show that the proposed test provides a lower detection error rate than the conventional K–S test.

This paper is organized as follows. In Section 2.1, we present the impulsive noise and signal models. In Section 3, we briefly introduces the K–S test and hypothesises for the test. In Section 4, we present the approximated distribution of the difference mentioned above on a certain amplitude, then the test is proposed by examining the distributions about observed amplitudes. In Section 6, the simulation results are presented in various environments. Finally, we conclude this paper with a brief summary, which is presented in Section 7.

## 2. Noise Models and Motivation for Noise Classification

### 2.1. Impulsive Noise and Received Signal Models

Consider a receiver with a received signal that only consists of noise samples. The *l*-th received sample at the receiver is written as

(1) y(l)=w(l),l=1,⋯,L,

where w(l) is the complex-valued noise sample at time *l* and *L* is the total number of the received samples by measurements. The noise samples {w(1),⋯,w(L)} are assumed to be additive white noise and their distribution is either Gaussian noise or impulsive noise. Among various impulsive noise models, Middleton class A noise and SαS noise models are widely accepted in the literature because their models are derived from the real physical mechanisms that generate disturbance in communication systems and agree well with physical phenomena [1,2].

The probability density function (PDF) of the instantaneous amplitude of Middleton class A noise is given as [1]

(2) $$f_{Z}\left(z\right)=\sum_{m=0}^{\infty }\frac{e^{-A}A^{m}}{m!}\frac{1}{\sqrt{2\pi \sigma_{m}^{2}}}e^{-\frac{z^{2}}{2\sigma_{m}^{2}}},$$

where the variance $\sigma_{m}^{2}$ is expressed as

(3) $$\sigma_{m}^{2}=\sigma^{2}\frac{\frac{m}{A}+\Gamma }{1+\Gamma },$$

where $\sigma^{2}$ is the average power of the noise. The parameter *A* is denoted as the impulsive index, which is the average number of *m* concerning the probability $\frac{e^{-A}A^{m}}{m!}$ [23]. Γ is called the Gaussian factor which is the ratio of the intensity of the independent Gaussian components and that of non-Gaussian components.

The other noise model is SαS noise model. A characteristic function of SαS noise is defined as

(4) $$\Phi_{Z}\left(jw\right)=e^{-{\left|\lambda w\right|}^{\alpha }+j\delta w},$$

where λ is the scale parameter larger than 0, δ is the location parameter with −∞<δ<∞, and α is the shape parameter with 0<α<2. Note that the PDF of the SαS noise is calculated by inverse Fourier transform, since the PDF does not have a closed form.

### 2.2. Motivation for Noise Classification

As mentioned earlier, noise classification (or an identification of noise statistics) of the surrounding environment is of prime importance for communication systems, since the optimal receiver design is based on the noise statistics to achieve optimal error performance. Note that most existing communication systems are designed to achieve optimal performance in an AWGN environment and those existing systems suffer significant performance loss in another noise environment.

**Figure 1:**
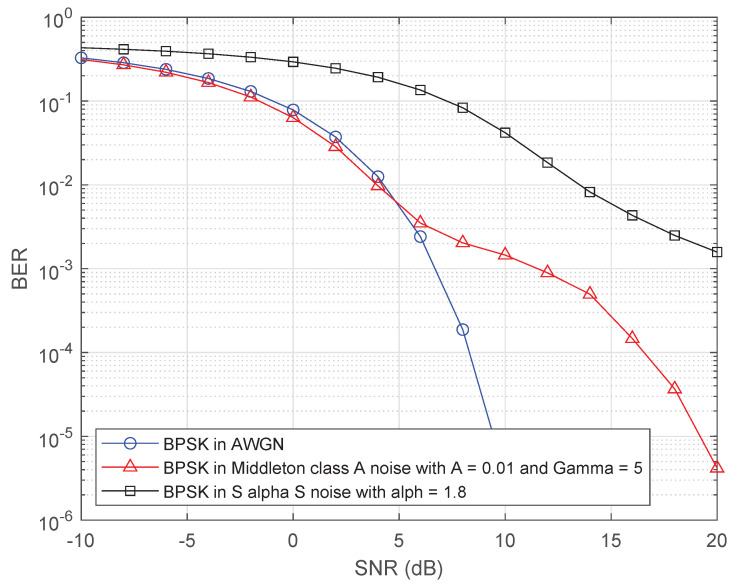
BER of BPSK in various noise environment such as AWGN, Middleton class A noise and SαS noise. Middleton class A noise parameters are set to (A,Γ)=(0.01,5) and SαS noise parameter is set to α=1.8.

Figure 1 shows a bit error rate (BER) performances of the conventional receiver with a binary phase-shift keying (BPSK) in impulsive noise and AWGN. In the case of an AWGN environment, the BER graph of BPSK is exponentially decreased as an SNR increases. When impulsive noise is present, however, the BER graph of BPSK shows a plateau due to the impulsive component. This clearly indicates that there is a performance loss for the conventional receiver when impulsive noise is present. Thus, a receiver needs to first classify the noise model in the given environment as accurately as possible in order to avoid or mitigate such performance loss. For this reason, it is essential to classify the noise model to decide a valid strategy since the communication method depends on the noise environment.

## 3. Conventional Kolmogorov–Smirnov Test

The K–S test is a non-parametric test based on the maximum difference between an empirical and a hypothetical cumulative distribution [24]. Failing to reject the null hypothesis shows that two data populations are drawn from the same distribution to a certain required level of significance. On the other hand, accepting the alternative hypothesis that they are from different distributions can be used. The hypotheses are stated as

(5) $$\begin{array}{cc} \; & H_{0}:F_{1}=F_{0}\\ \; & H_{1}:F_{1}\neq F_{0}, \end{array}$$

where $F_{0}$ is a hypothesized CDF and $F_{1}$ is an empirical CDF of the amplitude of received samples. Here, the hypotheses $H_{0}$ and $H_{1}$ are stated as a set of Gaussian amplitudes and a set of non-Gaussian amplitudes, respectively. The theoretical CDF of AWGN amplitude for the K–S test is computed as

(6) $$F_{0}\left(t\right)=P\left(w<t\right)=\int_{0}^{t}\frac{w}{2\sigma_{z}^{2}}e^{-\frac{w^{2}}{2\sigma_{z}^{2}}}dw=1-e^{-\frac{t^{2}}{2\sigma_{z}^{2}}},$$

where $\sigma_{z}^{2}=\frac{N_{0}}{2}$ and $N_{0}$ is a complex noise power. In the K–S test, the largest absolute difference between the empirical and the hypothesized CDFs is used as the goodness-of-fit statistic, which is given by

(7) $$D=\underset{0\leq t<\infty }{\sup}\left|F_{1}\left(t\right)-F_{0}\left(t\right)\right|,$$

where $F_{1}\left(x\right)$ is the value of the empirical CDF evaluated at *x*. For practical implementation with *N* measurement samples, where $t_{1},\ldots ,t_{N}$ are the amplitudes of those samples in ascending order, the difference between the empirical and the hypothesized CDFs for practical environments is computed as

(8) $$\hat{D}=\max_{1\leq i\leq N}\left|F_{1}\left(t_{i}\right)-F_{0}\left(t_{i}\right)\right|.$$

In addition, an empirical CDF from the measurement samples is computed as

(9) $$\begin{array}{c} F_{1}\left(t\right)=\frac{1}{N}\sum_{i=1}^{N}I\left(t_{i}\leq t\right), \end{array}$$

where **I**(·) is an indicator function that gives one if the condition is true for a sample, and zero otherwise and $t_{i}$ is the amplitude of the received sample in ascending order. The hypothesis $H_{0}$ is rejected at a significance level γ once $P(D>\hat{D})<\gamma$, where $P(D>\hat{D})$ is given as [18]

(10) $$P\left(D>\hat{D}\right)=Q\left(\left[\sqrt{N}+0.12+\frac{0.11}{\sqrt{N}}\right]\hat{D}\right),$$

where $Q\left(d\right)=2\sum_{m=1}^{\infty }{\left(-1\right)}^{m-1}e^{-2m^{2}d^{2}}$. [24] has demonstrated that Q(d) is convergent with thousands of *m* terms that may have large complexity for small error.

For detecting the presence of impulsive noise, the hypotheses in (5) are stated as

(11) $$\begin{array}{cc} \; & H_{0}:\left\{t_{1},\cdots ,t_{N}\right\}\mathrm{is}\;a\;\mathrm{set}\;\mathrm{of}\;\mathrm{AWGN}\;\mathrm{amplitudes},\\ \; & H_{1}:\left\{t_{1},\cdots ,t_{N}\right\}\mathrm{is}\;\mathrm{not}\;a\;\mathrm{set}\;\mathrm{of}\;\mathrm{AWGN}\;\mathrm{amplitudes}, \end{array}$$

## 4. Proposed Test

Although the K–S test is a common method to measure the goodness of a fit, [25] mentions that a test using K–S statistic to impulsive noise samples have poor performance since K–S statistic does not consider the characteristics of non-normal samples. This section proposes a test to detect the presence of impulsive noise considering the characteristics of impulsive noise. First, the idea behind the design of the proposed test is discussed. Then, an approximation of the threshold for the test hypothesis to satisfy target false alarm probability is presented.

### Design of the Proposed Test

**Figure 2:**
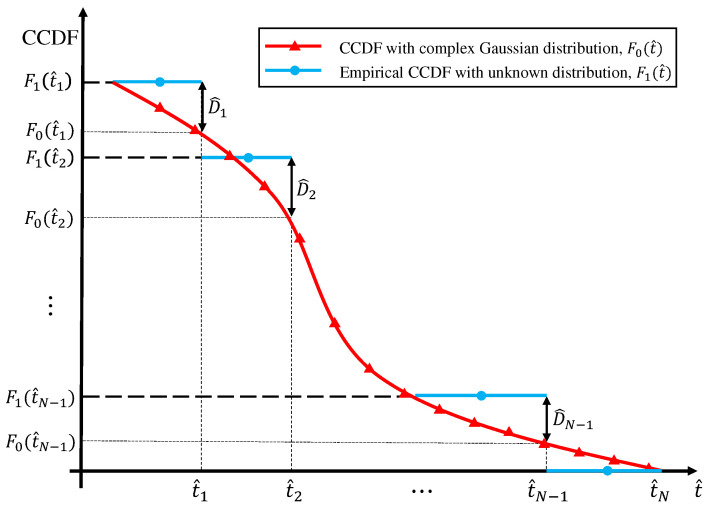
Plotting empirical CCDF with unknown distribution, theoretical CCDFs of complex Gaussian distribution and the differences ${\hat{D}}_{1},{\hat{D}}_{2},\cdots ,{\hat{D}}_{N-1}$ between them on ${\hat{t}}_{1},{\hat{t}}_{2},\cdots ,{\hat{t}}_{N-1}$ for the proposed test. *N* is the number of the received samples. $F_{0}\left({\hat{t}}_{i}\right)$ is the theoretical CCDF on ${\hat{t}}_{i}$ under the hypothesis. ${\hat{D}}_{i}$ is the differences between empirical and theoretical CCDFs on ${\hat{t}}_{i}$.

The discriminating feature for detecting the presence of impulsive noise is CCDF of the received signals are normalized amplitude since the bell-curve density of impulsive noise has a heavy-tails. The CCDF of the normalized amplitude of the received signal is compared with the reference CCDF of AWGN. Let us consider a measurement campaign that observes *N* noise measurement samples, where $t_{1},\cdots ,t_{N}$ are the amplitudes of those samples in ascending order, i.e., $t_{i}$ is the *i*-th smallest amplitude of the measurement samples. Since the value of $t_{i}$, the *i*-th smallest amplitude of the measurement samples, may not be the same for every measurement campaign, $t_{i}$ can be considered as a discrete random variable. Let ${\hat{t}}_{i}$ denote the actual value of the *i*-th smallest sample from a measurement campaign. Figure 2 illustrates the theoretical CCDF of complex Gaussian distribution and the empirical CCDF, which consists of those measurement samples with unknown distribution. The empirical CCDF is a decreasing step function that has the same number of steps as the number of samples because the number of measurement samples is finite. $D_{i}$ is the absolute difference between the two CCDFs for $t_{i}$ and is can be represented as

(12) $$D_{i}=|\left\{1-F_{1}\left(t_{i}\right)\right\}-\left\{1-F_{0}\left(t_{i}\right)\right\}\left|=\right|F_{0}\left(t_{i}\right)-F_{1}\left(t_{i}\right)|,$$

which is a random variable since $t_{i}$ is a random variable. When measurement samples are available, ${\hat{D}}_{i}$ is defined as the absolute value of the difference at ${\hat{t}}_{i}$ and is computed as

(13) $${\hat{D}}_{i}=\left|F_{0}\left({\hat{t}}_{i}\right)-F_{1}\left({\hat{t}}_{i}\right)\right|=\left|1-e^{-\frac{{\hat{t}}_{i}^{2}}{2\sigma_{z}^{2}}}-\frac{i}{N}\right|,$$

where $F_{0}\left({\hat{t}}_{i}\right)$ is computed by substituting *t* into ${\hat{t}}_{i}$ in (6) and $F_{1}\left({\hat{t}}_{i}\right)$ is given as $\frac{i}{N}$. That is, all the values of ${\hat{D}}_{i}$ are obtained by (13) where ${\hat{t}}_{1},\cdots ,{\hat{t}}_{N-1}$ in ascending order. Since the empirical CCDF on ${\hat{t}}_{N}$ equals to zero, ${\hat{t}}_{N}$ is not considered. Comparisons are carried out between the received signals CCDF and reference CCDF as shown in Figure 2. Unlike the conventional K–S test, the proposed test method considers all the values of ${\hat{D}}_{i}$ between the CCDFs in order to detect the presence of impulsive noise reliably. In addition, we employ a weighted sum of the probabilities of differences between the CCDFs for the proposed test since they are not equally weighted due to the characteristic of heavy-tails in impulsive noise.

To sum up, the proposed test is defined as

(14) $$S=\sum_{i=1}^{N-1}P_{i}w_{i}\overset{H_{0}}{\underset{H_{1}}{≶}}1-\gamma ,$$

where γ is the significance level, $P_{i}$ and $w_{i}$ will be discussed below. Finally, an algorithm based on (14) will be depicted. Firstly, $P_{i}$ in (14) is computed as

(15) $$P_{i}=\sum_{k\in K}P_{i,k},$$

where $P_{i,k}$ is the discrete probability density function of $D_{i}$ and *k* is the index of the discrete values of $D_{i}$. K is the set of those indices that satisfy the following condition:

(16) $$K=\{k\;|\;D_{i}\left(k\right)>{\hat{D}}_{i}\}.$$

$P_{i,k}$ can be obtained by *k* samples with the probability $F_{0}\left({\hat{t}}_{i}\right)$, and N−k samples with the probability $1-F_{0}\left({\hat{t}}_{i}\right)$, and the number of selecting *k* samples with the probability $F_{0}\left({\hat{t}}_{i}\right)$ among *N* samples is Nk. Therefore, the probability $P_{i,k}$ is computed as

(17) Pi,k=NkF0(t^i)k(1−F0(t^i))N−k.

**Figure 3:**
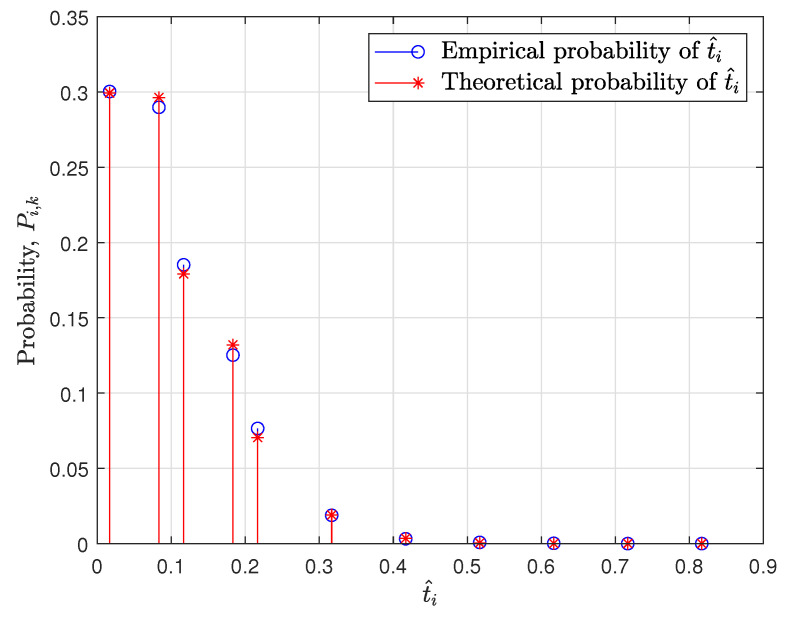
Plotting empirical probability $P_{i,k}$ with complex Gaussian distributed samples and theoretical probability by (17) on ${\hat{t}}_{i}$. ${\hat{t}}_{i}$ is 0.45 and $F_{0}\left({\hat{t}}_{i}\right)=0.8167$. For simulation, *N* equals to 10.

Figure 3 shows the empirical and theoretical probabilities of $P_{i,k}$ where ${\hat{t}}_{i}=0.45$ and ten samples are considered for the simulation above. It is clearly observed that the probabilities are well-matched.

## 5. Computation of Weight

As mentioned in the first section, the probabilities of the differences between the two CCDFs are not equally weighted to determine the presence of impulsive noise. Unlike the conventional K–S test, it is very important to give different weights for all of the received samples according to the difference in CCDF in our proposed algorithm. Therefore, it is significant to capture the CCDF difference more clearly between noise models. Impulsive noise is characterized by heavy-tails, which shows non-linearity in log(log(x)) and log scales. Whereas, AWGN is shown as linearity in log(log(x)) scales. Thus, we use a CCDF of log(log(x)) scale and log scale to calculate the weights using these differences.

**Figure 4:**
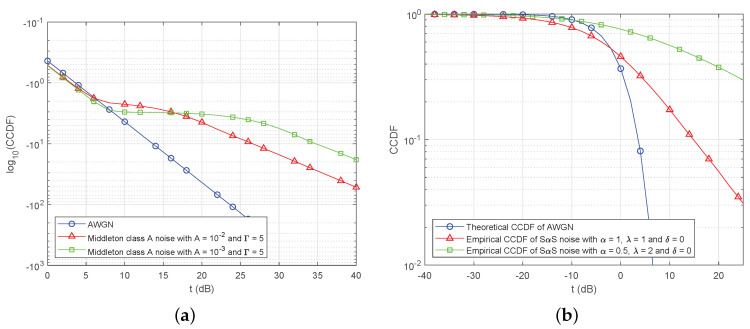
Illustration of theoretical CCDF of AWGN and empirical CCDFs of impulsive noise models. (**a**) Theoretical $\log_{10}\left(CCDF\right)$s of AWGN and Middleton class A noise with (A,Γ) = $\left(0.1,1\times 10^{-3}\right)$ and $\left(0.35,1\times 10^{-2}\right)$ on a log scale. (**b**) Theoretical CCDF of AWGN and empirical CCDFs of SαS noise with (α,λ,δ) = (1,1,0) and (0.5,2,0) on a log scale.

The weight for the proposed test is computed based on distributions of the noise. There are two noise models in terms of the distributions: Middleton class A noise and SαS noise models. Thus, we introduce the two weights for the impulsive noise models, respectively. First, Middleton class A noise model is considered. Let us assume that the parameters of noise model are not estimated, because the parameter estimation is carried out after detecting the presence of impulsive noise. Without the values of the parameters, it is impossible to do an exact calculation of the weight. Thus, a graphical approach of Middleton class A noise distribution is used based on the characteristics. Figure 4a illustrates CCDFs for a Middleton class A noise model and AWGN. The scale of the y-axis in Figure 4a shows an obvious difference between CCDFs of Middleton class A noise model and AWGN since the background component of Middleton class A noise and AWGN are illustrated as a straight line in the scale of y-axis in Figure 4a. The straight line can be used in order to compute a weight. Thus, the weight $w_{i,log-log}$ for $P_{i}$ becomes

(18) $$w_{i,log-log}=\frac{\left|\log_{10}\left(\frac{\log_{10}\left(F_{1}\left({\hat{t}}_{i}\right)\right)}{\log_{10}\left(F_{0}\left({\hat{t}}_{i}\right)\right)}\right)\right|}{\sum_{i=1}^{N-1}\left|\log_{10}\left(\frac{\log_{10}\left(F_{1}\left({\hat{t}}_{i}\right)\right)}{\log_{10}\left(F_{0}\left({\hat{t}}_{i}\right)\right)}\right)\right|},$$

Second, let us consider SαS noise model. Figure 4b shows CCDFs for SαS noise and AWGN. The CCDFs of SαS noise are plotted on log scale due to the fact that a slope of the CCDF is constant whereas the CCDF of AWGN is sharply decreasing in Figure 4b. Once the impulsiveness gets stronger, the linear curve with a constant slope of the CCDF gets higher. Owing to the linear curve in large amplitude, the logarithm function is suitable for giving more weight $P_{i}$ on a large amplitude due to the extremely low CCDF of AWGN. Since the difference of CCDFs between AWGN and SαS noise is remarkable on log-scale, for SαS noise, the weight of $P_{i}$ is

(19) $$w_{i,log}=\frac{\left|\log_{10}\left(F_{1}\left({\hat{t}}_{i}\right)\right)-\log_{10}\left(F_{0}\left({\hat{t}}_{i}\right)\right)\right|}{\sum_{i=1}^{N-1}\left|\log_{10}\left(F_{1}\left({\hat{t}}_{i}\right)\right)-\log_{10}\left(F_{0}\left({\hat{t}}_{i}\right)\right)\right|}.$$

By substituting (18) and (19) into $w_{i}$ in (14), weight sums $S_{log-log}$ and $S_{log}$ respectively corresponding to $w_{i,log-log}$ and $w_{i,log}$ are computed. Then, the hypothesis in (11) is determined by computing with 1−γ. For detecting the presence of impulsive noise, two weight sums are simultaneously used.

Finally, based on (14), (15), (18), and (19), the proposed algorithm for the test is stated in Algorithm 1.
**Algorithm 1** Proposed test
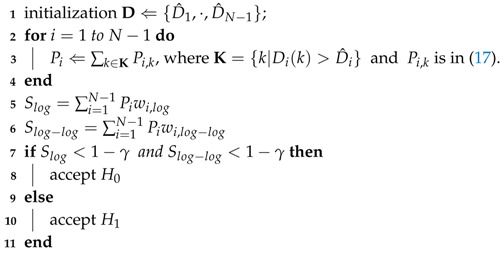


In Algorithm 1, D is a set of ${\hat{D}}_{i}$. The procedure is briefly explained along with the line numbers corresponding to each step as follows:

1. Initialization. Before the test, all CCDF differences ${\hat{D}}_{1},\cdots ,{\hat{D}}_{N-1}$ on ${\hat{t}}_{1},\cdots ,{\hat{t}}_{N-1}$ are computed (line 1).
2. $P_{i}$ can be computed by summing the probability $P_{i,k}$ in (15) based on (17) (line 2–4).
3. $S_{log}$ and $S_{log-log}$ are computed by multiplying $P_{i}$ in lines 2–4 and the weights $w_{i,log}$ and $w_{i,log-log}$, respectively (line 5–6).
4. Then, $S_{log}$ and $S_{log-log}$ are compared with 1−γ that is a threshold to determine if hypothesises are accepted or rejected (lines 7–11).

### Approximation of Threshold for the Proposed Test

**Figure 5:**
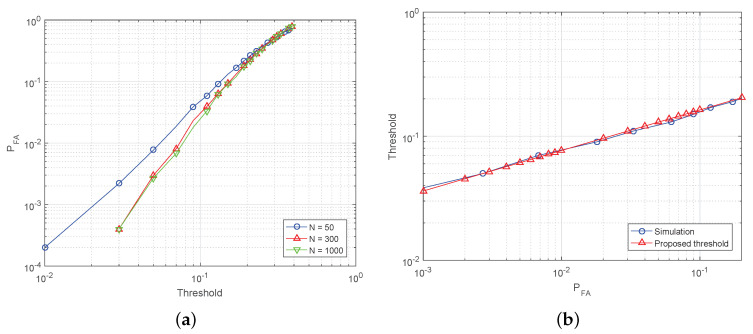
Illustration of theoretical CCDF of AWGN and empirical CCDFs of impulsive noise models. (**a**) Plotting threshold versus $P_{FA}$ in N= 50, 300 and 1000. (**b**) Plotting $P_{FA}$ versus simulation results of threshold in N= 1000 and derived threshold in (25).

The test to detect the presence of impulsive noise is proposed in the above subsection. However, a threshold to satisfy a target $P_{FA}$ is not defined. Thus, the threshold to satisfy $P_{FA}$ is derived. The threshold of the proposed test is approximated due to its complexity. To approximate the threshold, the asymptotic performance of the proposed test is analyzed. Figure 5a shows $P_{FA}$ with N= 50, 300 and 1000 to show asymptotic performance of the test. It is clear that the false alarm probability $P_{FA}$ of the proposed test is improved with a higher *N* but there is no considerable improvement when the number of samples *N* exceeds 1000. Therefore, we can obtain the approximation of the threshold on the $P_{FA}$ in *N* = 1000. Value of the threshold in the range between 0.05 to 0.2 is considered in Figure 5a, because the curve in Figure 5a can be modelled as a linear curve. The x-axis and y-axis of the curve are log-scaled in Figure 5a and the relation between log-scaled x-axis and y-axis becomes

(20) $$\log_{10}\left(y\right)=a\log_{10}\left(x\right)+b,$$

where *a* is a slope of the linear function and *b* is a y-intercept. Thus, the relation between $P_{FA}$ and threshold in Figure 5a is given as

(21) $$\log_{10}\left(P_{FA}\right)=a\log_{10}\left(\gamma \right)+b.$$

To determine *a* and *b*, two pairs of $\left(\log_{10}\left(P_{FA}\right),\log_{10}\left(\gamma \right)\right)$ are required and Table 1 shows the pairs obtained from simulation results. Once the two pairs of $\left(\log_{10}\left(P_{FA}\right),\log_{10}\left(\gamma \right)\right)$ are considered, *a* is computed as

(22) $$\begin{array}{cc} a & =\frac{\log_{10}\left(P_{FA,\gamma =0.2}\right)-\log_{10}\left(P_{FA,\gamma =0.05}\right)}{\log_{10}\left(0.2\right)-\log_{10}\left(0.05\right)}\\ \; & =\frac{\left(-0.6607)-(-2.5686\right)}{-0.6778-(-1.3010)}=3.0612. \end{array}$$

Also, *b* is obtained as

(23) $$\begin{array}{cc} b & =\log_{10}\left(P_{FA,\gamma =0.05}\right)-a\cdot \log_{10}\left(0.05\right)\\ \; & =-2.5686-3.0612\times (-1.3010)=1.4141. \end{array}$$

By substituting *a* in (22) and *b* in (23) into *a* and *b* in (21), the relation is decided as

(24) $$\log_{10}\left(P_{FA}\right)=3.0612\log_{10}\left(\gamma \right)+1.4141.$$

Therefore, the threshold γ is determined as

(25) $$\gamma =P_{FA}^{0.3267}\cdot 10^{-0.4619}.$$

Figure 5b shows the simulation results and proposed threshold in (25) to satisfy target $P_{FA}$. As shown in the figure, (25) and simulated thresholds are well matched.

## 6. Results and Discussion

In this section, simulation results are provided to evaluate the performance of the proposed test methodology, especially the miss detection probability performance for classification an impulsive noise model under various false alarm probability. For the extensive simulation, the false alarm probability is given as

(26) $$P_{FA}=P\left(\mathrm{Decision}\;\mathrm{of}\;H_{1}|H_{0}\;\mathrm{is}\;\mathrm{true}\right),$$

and the miss detection probability is shown as

(27) $$P_{MD}=P\left(\mathrm{Decision}\;\mathrm{of}\;H_{0}|H_{1}\;\mathrm{is}\;\mathrm{true}\right).$$

where $P_{D}$ is the detection probability.

For simulations, complex impulsive noise samples are generated based on the Middleton class A model in (2) and SαS model in (4), respectively, where the real and the imaginary components are dependent and uncorrelated. For the Middleton class A, the model is characterized by the parameters *A* and Γ such that the impulsiveness of the noise can be represented with *A* and Γ. When *A* is increasing, Middleton class A noise becomes more likely to be Gaussian noise and, conversely, a low value of *A* represents highly structured impulsive noise especially when A≪1 and ΓA≪1 [26,27]. Therefore, in order to effectively compare the performance of the proposed test method, we set *A* and Γ to 0.01 and 5, respectively, for the impulsive noise model. The SαS model is characterized by the parameter α, and if the value of α is small that implies the stronger the impulsive property. In general, the value of α falls into between 1 and 2 in a practical environment [28]. Thus, we consider the SαS model with α equals to 1.8 in this paper.

Receiver operating characteristic (ROC) analysis is widely used in signal processing and communications systems to evaluate the detection performance, where it exploits two-dimensional curves plotted by detection probability against false alarm probability [29]. Note that we adopt the $P_{MD}$ as a performance metric instead of $P_{D}$, thus the ROC curve is represented with $P_{MD}$ and $P_{FA}$, in this paper.

**Figure 6:**
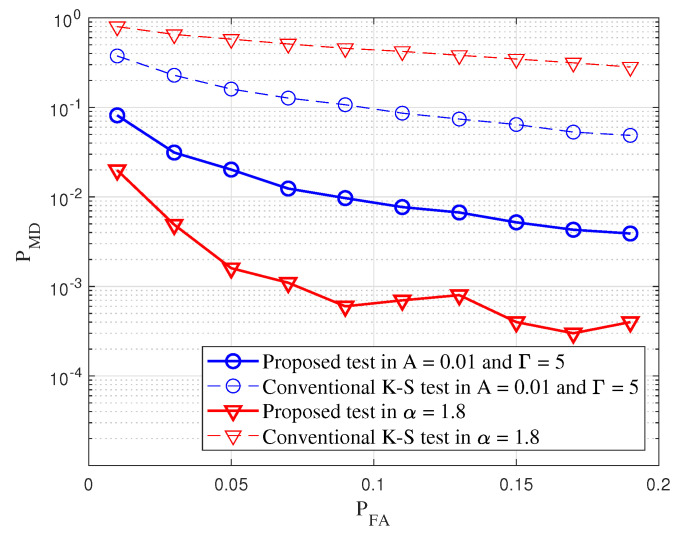
Comparison of $P_{MD}$ of the proposed test and the conventional K–S test in various impulsive noise environment. The parameter of Middleton class A noise is $A=10^{-2}$ and Γ=5. Those of SαS noise equals to α=1.8, δ=0, and λ=1/4. *N* equals to 500.

Figure 6 shows a comparison of $P_{MD}$ of the conventional K–S test and the proposed test in Middleton class A noise with respect to false alarm probability where the number of observed samples are 500. Here, it was observed that the proposed test has a lower miss detection probability than the K–S test in the various impulsive noise environments. In particular, it can be easily observed that the proposed test outperforms the K–S test by providing approximately 30% lower miss detection probability at a $P_{FA}=0.01$ for the Middleton class A noise environment. Furthermore, in the case of SαS noise model, the CCDF of the impulsive noise model is similar to the Gaussian noise. It means that the difference of CCDF of those is not considered at any ${\hat{t}}_{i}$. Thus, the detection performance of the conventional K–S test to SαS is poor. Note that the K–S test has a higher miss detection probability under various false alarm probability because the K–S test does not enough capture the characteristics of impulsive noise compared with the proposed test. On the other hand, compared with the K–S test in the SαS noise model, the miss detection probability gain of the proposed test over the K–S test is significant even at low false alarm probability.

In summary, the conventional K–S test uses the maximum difference between CCDFs of received samples and the assumed distribution when the distribution of received samples follows the distribution of impulsive noise. However, the maximum difference may correspond to Gaussian component that is not the characteristic of impulsive noise when an impulsive property is stronger. Unlike the conventional K–S test, the proposed test considers all amplitudes of the received samples and computes differences of CCDFs between the assumed distribution and the received samples. Besides, the proposed test improves the classification performance by utilizing the characteristics of impulsive noise that it gives more weight to amplitudes corresponding to the characteristics of impulsive noise than weight for the other amplitudes corresponding to the Gaussian component. Through the simulation results, it was confirmed that the $P_{MD}$ performance of our proposed method was improved by at least 28% and 87% for Middleton class A and SαS models, respectively, compared to the existing conventional K–S test performance.

## 7. Conclusions

This paper proposes a test to detect the presence of impulsive noise. The proposed method is designed based on a weighted sum of all differences between CCDFs using thresholds. To improve detection performance, different weights are proposed according to the Middleton class A and SαS model, respectively. Finally, a threshold to satisfy target false alarm probability is obtained by analyzing an asymptotic false alarm probability. In particular, a significant highlight of our proposed test method is that it operates on a high detection probability with low false alarm probability; it can be used in various cellular networks, for example, a vehicular network where an impulsive noise exists.

## Figures and Tables

**Table 1:** Two pairs of $\left(\log_{10}\left(\gamma \right),\log_{10}\left(P_{FA}\right)\right)$ in N=1000.

$\log_{10}\left(\gamma \right)$	−1.3010	−0.6778
$\log_{10}\left(P_{FA}\right)$	−2.5686	−0.6607

## Abbreviations

The following abbreviations are used in this manuscript:

CCDF	Complementary distribution function
SαS	Symmetric α stable
